# Association of Serum Ferritin With Severity of Disease in Real-Time Reverse Transcription-Polymerase Chain Reaction Negative COVID-19 Patients

**DOI:** 10.7759/cureus.41065

**Published:** 2023-06-28

**Authors:** Boyina V Sai Bharath, Promod K Tudu, Subhash C Dash, Nalinikanta Sahoo

**Affiliations:** 1 Department of Neurology, Jawaharlal Nehru Medical College, Karnataka, IND; 2 Department of General Medicine, Institute of Medical Sciences and SUM Hospital, Siksha ‘O’ Anusandhan Deemed to be University, Bhubaneswar, IND; 3 Department of General Medicine, Institute of Medical Sciences and SUM Hospital, Siksha 'O' Anusandhan Deemed to be University, Bhubaneswar, IND

**Keywords:** serum ferritin, rt-pcr negative, corads-5, inflammatory markers, covid-19, severe disease

## Abstract

Background: Coronavirus disease 2019 (COVID-19) is still causing disastrous effects in various parts of the world through recurring waves. Real-time reverse transcription polymerase chain reaction (RT-PCR)-negative COVID-19 is particularly challenging as these patients are less likely to receive treatment and more likely to progress to severe disease. Thus, it is imperative to find markers that can predict the severity of disease at an early stage. The objective of the present study was to analyze the association of ferritin levels with severe disease in RT-PCR-negative COVID-19 patients.

Methods: A prospective cross-sectional analytical study was conducted in adults with COVID-19 pneumonia with a negative RT-PCR test from October 2020 to September 2021. Hematologic, biochemical, and inflammatory parameters were investigated within 24 h of hospitalization. Demographic, clinical, and laboratory findings were compared between patients with and without severe disease.

Results: A total of 220 patients were included. The mean age of the study participants was 47.3 ± 14.2 years, and 55.5% (n=122) were male. C-reactive protein, D-dimer, and ferritin levels were significantly higher in patients with severe disease (p<0.01). Receiver operating characteristic curve analyses were performed, and ferritin was found as significant predictor of severe disease (area under the curve=0.642, p<0.001).

Conclusion: Early analysis of ferritin can predict the severity of disease in COVID-19 patients, irrespective of the RT-PCR status.

## Introduction

The coronavirus pandemic has caused turmoil all over the world. It is still causing disastrous effects in various parts of the world through recurring waves. Coronavirus disease 2019 (COVID-19) has diverse clinical presentations. In fact, the clinical spectrum of COVID-19 is vast, ranging from asymptomatic or mild upper respiratory infections to severe pneumonia with respiratory or multi-organ failure [1-3]. The routine method for detection of severe acute respiratory syndrome coronavirus 2 (SARS-CoV-2) is real-time reverse transcriptase polymerase chain reaction (RT-PCR) on oropharyngeal and nasopharyngeal swab samples, and it is considered the gold standard. However, it becomes a challenge for clinicians while managing patients with typical clinical manifestations of COVID-19 but negative RT-PCR results. A systematic review of the real-world diagnostic sensitivity of RT-PCR (nasopharyngeal swab) reports that up to 33% of COVID-19 patients may have false-negative results despite a compatible clinical illness, consistent thoracic imaging and/or subsequent positive antibodies to COVID-19 [4,5]. Further, Parmar et al. identified a cohort of repeatedly RT-PCR-negative patients, despite presenting with typical COVID-19 signs and/or symptoms [6]. Moreover, various studies demonstrate that RT-PCR-negative COVID-19 is common among patients admitted to hospitals with clinical characteristics and severity comparable to RT-PCR-confirmed COVID-19 [5-7]. RT-PCR-negative COVID-19 patients are half as likely to get the treatment than RT-PCR-confirmed patients. Parmar et al. [6] further observed that 43% of RT-PCR-negative COVID-19 patients with mild-moderate disease progressed to severe hypoxic disease, thereby representing a window for interventions to reduce the disease's progression. Thus, it is imperative to find the marker that can predict the severity of illness early.

The severity and mortality of COVID-19 have been attributed to an inflammatory cytokine storm. The cytokine storm is characterized by the excess release of inflammatory cytokines as a dysfunctional immune response to SARS-CoV-2 infection [8,9]. Ferritin contributes to the cytokine storm through direct immune-suppressive and pro-inflammatory effects, especially in the hyper-ferritinemia state due to the infection [10]. A study by Bozkurt et al. suggested that ferritin level at admission in hospitalized patients with COVID-19 may predict the severity of the disease [11]. Similarly, a Brazilian study by Lino et al. concluded that a high ferritin level at the admission of COVID-19 patients is an independent predictor of mortality [12]. Further, a meta-analysis involving 10,614 COVID-19 patients concluded that ferritin levels were significantly higher in patients with severe disease and associated with a poor prognosis [13]. But these studies are limited to RT-PCR-confirmed COVID-19 patients. We, therefore, aimed to study whether ferritin levels can predict the severity of disease in RT-PCR-negative COVID-19 patients.

## Materials and methods

This cross-sectional analytical study was conducted at the Institute of Medical Sciences & SUM Hospital, a tertiary care referral hospital in Bhubaneswar, India. Patients presented with symptoms of COVID-19 were primarily admitted through the emergency department after performing RT-PCR on nasopharyngeal or oropharyngeal swab samples. The study was initiated with a prospective design that enrolled patients admitted with signs and symptoms of COVID-19, but the RT-PCR test was negative. We performed high-resolution computed tomography (HRCT) of the chest in these patients, and an independent radiologist examined the images for COVID-19 Reporting and Data System (CO-RADS) assessment [14]. CO-RADS 1 implies normal CT or CT findings of unequivocal non-infectious etiology, whereas CO-RADS 5 implies CT findings are typical for COVID-19. CO-RADS 6 indicates RT-PCR-confirmed COVID-19. Patients of the CO-RADS 5 category were included in the study in a consecutive sample of cases from October 2020 to September 2021. The time window of the study established the sample size, and no sample size calculation was performed. Patients <18 years of age, those with evidence of diagnoses other than COVID-19 and a history of cirrhosis of liver, liver failure or hepatoma, were excluded from the study. The study was conducted after getting approval from the Institutional Ethics Committee (Ref. No/DRI/IMS.SH/SOA/21/042), and written informed consent was obtained from all participants.

Data collection

All the participants were clinically evaluated, and demographic data, comorbidities (such as asthma, chronic obstructive pulmonary disease, diabetes mellitus, hypertension, coronary heart disease, and chronic kidney disease), vital signs, and clinical and laboratory findings were recorded. Besides routine hematologic and biochemical parameters, laboratory investigations included c-reactive protein (CRP), D-dimer, and serum ferritin. These studies were performed within 24 h of hospital admission by automated methods using Sysmex XN-300 (Sysmex America Inc., Illinois, USA), Cobas Integra 400 plus (Roche Diagnostics, Basel, Switzerland), and Cobas e 411 (Roche Diagnostics, Basel, Switzerland). Serum ferritin was estimated by the chemiluminescence immunoassay (ECLIA) method. Severe disease was defined in patients having dyspnea (respiratory rate (RR) >24/min), low oxygen saturation (SpO_2_ ≤93%) in room air, respiratory distress or requiring mechanical ventilation, and/or admission to intensive care unit (ICU) [15,16].

Statistical analysis

The statistical analysis was performed using SPSS version 20.0 (IBM Corp., Armonk, NY). The results are presented as frequencies and percentages or mean with standard deviation. Participants were categorized into those with or without severe disease. We compared the categorical variables between the two groups, using Chi-square and Fisher exact tests as applicable. An independent t-test was used to assess the differences between continuous variables. Further, we performed the receiver operating characteristic curve (ROC) to evaluate the predictability of the severity of the disease and calculated the area under the curve (AUC). The cut-off value was determined as the maximum value giving the best balance between sensitivity and specificity. The two-tailed p-value of <0.01 was considered a statistically significant difference.

## Results

A total of 220 adult patients were enrolled in this study. The mean age of the study population was 47.3 ± 14.2 years, and 55.5% (n=122) were male. The most common presenting symptom was fever (83.6%), followed by cough (79.5%) and shortness of breath (47.3%). The mean oxygen saturation in the participants was 90.5 ± 7.2% and 51.4% (n=113) of participants were found to have severe disease. Table 1 summarizes the baseline characteristics of the participants.

**Table 1:** Baseline characteristics of the study participants (n=220). SD: standard deviation; BP: blood pressure; SpO_2_: oxygen saturation; ICU: intensive care unit; CT: computerized tomography.

Parameter	n (%)
Sex, male	122 (55.5)
Age (years), mean ± SD	47.3 ± 14.2
Age >60 years	49 (22.3)
Current smoker	19 (8.6)
Patients with comorbidities	115 (52.3)
Presenting symptoms	
Fever	184 (83.6)
Shortness of breath	104 (47.3)
Myalgia	65 (29.5)
Cough	175 (79.5)
Diarrhea	18 (8.2)
Sore throat	61 (27.7)
Headache	21 (9.5)
Fatigue	79 (35.9)
Duration of symptoms till PCR (days), median (IQR)	5.5 (5-7)
CT severity score, mean ± SD	14.3 ± 4.0
Vital signs	
Temperature (°C), mean ± SD	37.8 ± 0.6
Heart rate (beats/min), mean ± SD	89.8 ± 16.9
Respiratory rate (breaths/min), mean ± SD	20.1 ± 6.5
SpO_2 _(%), mean ± SD	90.5 ± 7.2
Systolic BP (mm Hg), mean ± SD	115.1 ± 17.2
Diastolic BP (mm Hg), mean ± SD	70.5 ± 10.8
Admission	
ICU	53 (24)
Ward	167 (75.9)
Severe cases	113 (51.4)

A comparison of demographic and biochemical parameters between the participants according to the severity of the disease is shown in Table 2. Demographic parameters like age, gender, and the frequency of comorbid conditions were not significantly different between patients with severe disease and non-severe disease. However, serum ferritin levels were significantly higher in patients with severe disease (693.16 ± 290.15 vs. 549.11 ± 277.76; p<0.001). CRP and D-dimer levels were also higher in severe cases (p<0.01).

**Table 2:** Demographic and biochemical parameters of the participants according to the severity of the disease. COPD: chronic obstructive pulmonary disease; CKD: chronic kidney disease; CHD: coronary heart disease; WBC: white blood cell; AST: aspartate aminotransferase; ALT: alanine aminotransferase; LDH: lactate dehydrogenase.

Parameter	Non-severe	Severe	p-value
Age (years)	46.95 ± 14.13	47.73 ± 14.41	0.689
Male sex	64 (59.8)	58 (51.3)	0.224
Age >60 years	23 (21.5)	26 (23)	0.906
Current smoker	8 (7.5)	11 (9.7)	0.635
Hypertension	47 (43.9)	52 (46)	0.787
Diabetes mellitus	37 (34.5)	43 (38)	0.674
Asthma	6 (5.6)	9 (7.9)	0.596
COPD	7 (6.5)	12 (10.6)	0.341
CKD	10 (9.3)	15 (13.2)	0.401
CHD	17 (15.8)	23 (20.3)	0.485
Hemoglobin (g/dL)	12.16 ± 1.18	11.92 ± 1.25	0.149
WBC (×10³ cells per μL)	6.3 ± 1.63	7.0 ± 2.11	0.010
Platelets (×10³ cells per μL)	216.36 ± 64.89	212.52 ± 61.96	0.654
Bilirubin (mg/dL)	0.72 ± 0.21	0.75 ± 0.25	0.322
AST (U/L)	57.36 ± 37.85	63.52 ± 36.00	0.217
ALT (U/L)	33.10 ± 12.77	35.79 ± 14.11	0.141
Albumin (g/dL)	3.06 ± 0.26	3.03 ± 0.28	0.465
Creatinine (mg/dL)	1.44 ± 0.52	1.52 ± 0.52	0.295
Ferritin (μg/L)	549.11 ± 277.76	693.16 ± 290.15	<0.001
C-reactive protein (mg/dL)	9.66 ± 1.75	10.26 ± 1.51	0.008
LDH (U/L)	686.86 ± 166.38	699.76 ± 171.75	0.573
D-dimer (μg/mL)	1.45 ± 0.52	1.61 ± 0.34	0.009
Mortality	0 (0)	27 (23.8)	<0.001

**Figure 1:**
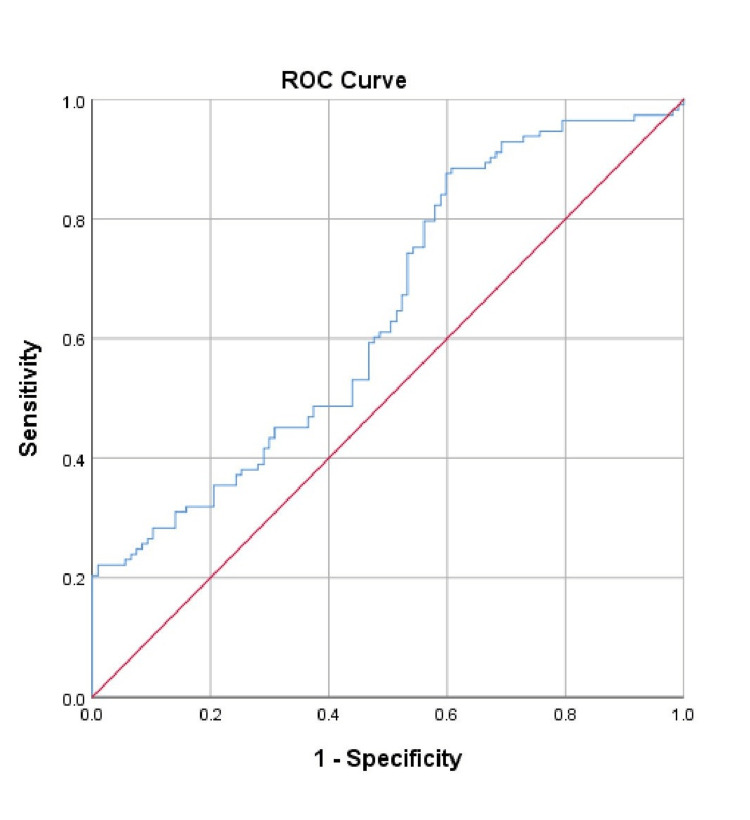
ROC curve for ferritin levels. ROC: receiver operating characteristic curve.

ROC curve analyses were performed for ferritin, CRP, and D-dimer levels to predict disease severity. ROC curves showed lower AUC values and no statistical significance (p=0.01) for CRP and D-dimer, whereas ferritin presented a higher AUC value of 0.642 with statistical significance (p<0.001). Sensitivity and specificity for different cut-off values of serum ferritin have been shown in Figure 1. As measured by the area under the curve (AUC) for ferritin, the 95% confidence interval was 0.569 to 0.714 for the severity of illness. Sensitivity and specificity were 54% and 53.3%, respectively, at a ferritin cut-off value of 608.9 μg/L.

## Discussion

The present study was conducted in an academic tertiary care center during the pandemic with intervening first (March 2020 to January 2021) and second (March 2021 to June 2021) waves in India. The study included RT-PCR-negative COVID-19 patients, and inflammatory markers were sent on the first day of admission. The median age of the study participants was 48 years, with a male preponderance comparable to various studies on COVID-19 patients [17,18]. The most common presenting symptom was fever, followed by cough and breathlessness, and the most prevalent comorbidity was hypertension in our cohort, like in other studies [11,18].

Our study showed a significant increase in inflammatory markers like CRP, serum ferritin, and D-dimer levels in patients with severe disease. CRP levels are a non-specific marker of high levels of inflammation. Its level is also implicated in the severity of illness in COVID-19 patients [17]. A meta-analysis including 16 studies revealed CRP levels were positively related to the severity of COVID-19 in all except one study [19]. Similarly, our study showed significantly increased CRP levels in patients with severe disease.

Increased D-dimer level indicates the development of a hypercoagulable state in COVID-19 infection. Thus, it is a significant biomarker that prompts immediate intervention to prevent further complications. In an animal study, Yu et al. [20] found increased urokinase activity in COVID-19 infection and the conversion of plasminogen to plasmin resulting in hyperfibrinolysis and the development of acute respiratory distress syndrome (ARDS). Various studies have also reported that D-dimer level increases with the severity of illness in COVID-19 patients [11,21]. In our research, the D-dimer level was also markedly increased in severe cases with a statistically significant difference.

Increased ferritin levels result from excessive inflammation as inflammatory cytokines stimulate hepatocytes, Kupffer cells, and macrophages to secrete ferritin [22]. It also plays a pathogenic role in the inflammation process by promoting the expression of multiple pro-inflammatory mediators. Ferritin can be a direct indicator of cellular damage, especially when its level exceeds 600 μg/L, reflecting a direct link between ferritin production and organ damage [23]. Further, ferritin contributes to the cytokine storm causing potential acute respiratory distress syndrome and systemic organ failure [9,10]. Liu et al. [24] observed significantly elevated levels of ferritin in patients with severe COVID-19 and concluded that serum ferritin levels were closely related to the severity of COVID-19. Further, Zhou et al. [25] reported that increased ferritin levels were associated with the worsening of COVID-19 in their study. Moreover, a meta-analysis including a total of 52 studies and 10614 COVID-19 patients revealed the role of ferritin in indicating a severe disease and a mortality risk in COVID-19 patients [13]. Our study, too, observed significantly higher ferritin levels in patients with severe illness compared with non-severe illness. In our research, we also evaluated the predictability of CRP, D-dimer, and ferritin levels for the severity of illness. We found ferritin as the only significant predictor of severe disease (p<0.001) in our cohort of COVID-19 patients. Bozkurt et al. and Cao et al. also reported serum ferritin levels as predictors of the severity of illness in patients with COVID-19 in their respective studies [11,26]. Our study observed a ferritin level of ≥ 608.9 μg/L for predicting disease severity with a sensitivity of 54% and a specificity of 53.3% (AUC=0.642). In a study including 330 COVID-19 patients, Bennouar et al. [27] reported a similar AUC of 0.63 for predicting severe disease. Feld et al. [28] also reported an almost similar AUC of 0.68 for severity in a cohort of 942 COVID-19 patients.

Our study has some limitations. First, it is a single-center study including only RT-PCR-negative COVID-19 patients; thereby, selection bias is possible. However, selecting patients with clinical and radiological signs typical of COVID-19 during the pandemic, with intervening first and second waves, might minimize the bias. Second, the study included only hospitalized cases, which may impact the generalizability of the study's findings. Third, although the study was prospective, it did not evaluate serial ferritin levels and the prediction of changes in ferritin values on the outcome.

## Conclusions

In conclusion, our study findings support the hypothesis that ferritin levels might be crucial in indicating the severity of disease in COVID-19 patients. Assessment of admission ferritin may serve as an easy-to-use predictor for the severity of disease in COVID-19 patients, irrespective of their RT-PCR status. However, further studies are needed to validate the findings of our study.
